# Mutational spectrum of autosomal recessive limb-girdle muscular dystrophies in a cohort of 112 Iranian patients and reporting of a possible founder effect

**DOI:** 10.1186/s13023-020-1296-x

**Published:** 2020-01-14

**Authors:** Marzieh Mojbafan, Reza Bahmani, Samira Dabbagh Bagheri, Zohreh Sharifi, Sirous Zeinali

**Affiliations:** 1 Department of Medical Genetics and Molecular Biology, Faculty of Medicine, Iran University of Medical Sciences (IUMS), Shahid Hemmat Highway, Tehran, Iran; 2 Department of Medical Genetics, Ali-Asghar Children’s Hospital, Zafar St., Shahid Modarres Highway, Tehran, Iran; 3 Student Research Committee, Faculty of Medicine, Iran University of Medical Sciences, Tehran, Iran; 4 Dr. Zeinali’s Medical Genetics Laboratory, Kawsar Human Genetics Research Center, Tehran, Iran; 5 Department of Genetics, Faculty of Advanced Science and Technology, Tehran Medical Sciences, Islamic Azad University, Tehran, Iran; 6 Department of Molecular Medicine, Biotechnology Research Center, Pasteur Institute of Iran, No. 69, Pasteur Ave, Tehran, Iran

**Keywords:** Limb-girdle muscular dystrophy, Autozygosity mapping, Founder effect, Novel mutations, Iran

## Abstract

**Background:**

Limb-girdle muscular dystrophies are a group of genetically heterogeneous diseases that are inherited in both autosomal dominant (LGMDD) and autosomal recessive forms (LGMDR), the latter is more common especially in populations with high consanguineous marriages like Iran. In the present study, we aimed to investigate the genetic basis of patients who are suspicious of being affected by LGMDR.

DNA samples of 60 families suspected of LGMD were extracted from their whole blood. Four short tandem repeat (STR) markers for each candidate genes related to LGMD R1 (calpain3 related)- R6 (δ-sarcoglycan-related) were selected, and all these 24 STRs were applied in two sets of multiplex PCR. After autozygosity mapping, Sanger sequencing and variant analysis were done. Predicting identified variants’ effect was performed using in-silico tools, and they were interpreted according to the American College of Medical Genomics and Genetics (ACMG) guideline. MLPA was used for those patients who had large deletions.

Fresh muscle specimens were taken from subjects and were evaluated using the conventional panel of histochemical stains.

**Results:**

forty out of sixty families showed homozygote haplotypes in *CAPN3, DYSF, SGCA,* and *SGCB* genes. The exons and intron-exon boundaries of the relevant genes were sequenced and totally 38 mutations including *CAPN3* (*n* = 15), *DYSF* (*n* = 9), *SGCB* (*n* = 11), and *SGCA* (*n* = 3) were identified. Five out of them were novel. The most prevalent form of LGMDs in our study was calpainopathy followed by sarcoglycanopathy in which beta-sarcoglycanopathy was the most common form amongst them. Exon 2 deletion in the *SGCB* gene was the most frequent mutation in this study.

We also reported evidence of a possible founder effect in families with mutations in *DYSF* and *SGCB* genes. We also detected a large consanguineous family suffered from calpainopathy who showed allelic heterogeneity.

**Conclusions:**

This study can expand our knowledge about the genetic spectrum of LGMD in Iran, and also suggest the probable founder effects in some Iranian subpopulations which confirming it with more sample size can facilitate our genetic diagnosis and genetic counseling.

## Background

Limb-girdle muscular dystrophies are a group of genetically heterogeneous disorders in which mainly the pelvic and shoulder girdle muscles are progressively involved [1]. They are inherited in both autosomal dominant (LGMDD) and autosomal recessive forms (LGMDR), the latter is more common [2], and is more observed in populations with high consanguineous marriages [3].

Twenty-six types of LGMDR have been identified so far in which LGMDR1 calpain3-related (LGMD2A, calpainopathy), LGMDR2 dysferlin-related (LGMD2B, dysferlinopathy^1^), and sarcoglycanopathies including LGMDR5 γ-sarcoglycan-related (LGMD2C), LGMDR3 α-sarcoglycan-related (LGMD2D), LGMDR4 β-sarcoglycan-related (LGMD2E), and LGMDR6 δ-sarcoglycan-related (LGMD2F), are the most common kinds of LGMDRs and are caused by mutations in *CAPN3*, *DYSF, SGCG (*γ-sarcoglycan)*, SGCA (*α-sarcoglycan)*, SGCB (*β-sarcoglycan)*,* and *SGCD (*δ-sarcoglycan) genes respectively [4].

Calpain-3 plays an essential role in sarcomere remodeling [5]. It is an enzyme that can be in active or inactive forms by its proteolytic activity. It can also cleave some cytoskeletal and myofibrillar proteins [6]. This protein is encoded by the *CAPN3* gene that is located on 15q15.1 and consists of 24 exons [7]. The *DYSF* gene is located on 2p13.2 and contains 55 exons. Dysferlin is a transmembrane protein, which takes part in sarcolemmal resealing, differentiation and regeneration of muscles, and is involved in stabilizing stress-induced calcium signaling in the transverse tubule. This protein is mainly expressed in skeletal muscle, heart, and kidney [8–11]. *SGCA* gene is on 17q21, which is composed of 10 exons. *SGCB* and *SGCG* genes located on 4q12 and 13q12, and have 6 and 8 exons, respectively. *SGCD* gene locus is on 5q33.2 and consists of 9 exons. In skeletal muscle, these sarcoglycans compose heterotetramers in the sarcolemma. Sarcoglycans form dystrophin-glycoprotein complex (DGC) along with other proteins that connect the muscle fiber cytoskeleton to the extracellular matrix [12].

Autozygosity Mapping uses the fact that patients who born from consanguineous marriages probably inherit two recessive copies of a mutant allele from a common ancestor. The purpose of this method is to search for regions with homozygosity, which can vary from a few to several megabases in the patient’s DNA. This way will be followed by identifying the region that carries a mutated gene involved in rare recessive traits [13]. Autozygosity Mapping is a powerful approach for gene tracking of autosomal recessive diseases in consanguineous families like Iran [14], and it can be the right choice for gene mapping in heterogeneous diseases such as LGMDs.

This study aims to investigate disease-causing mutations of genes responsible for LGMDR1 calpain3 related- R6 δ-sarcoglycan-related in 60 families who are suspicious of being affected by LGMDRs by autozygosity mapping followed by Sanger sequencing.

## Results

### Patient population and LGMD diagnosis

We evaluated 60 families for different mutations in our center. Most affected individuals born to consanguineous marriages and 40 out of 60 families with 112 patients showed homozygote haplotypes in *CAPN3, DYSF, SGCA,* and *SGCB* genes. Table 1 shows the clinical and paraclinical features of the available patients.

**Table 1:** clinical features and mutations observed in the available patients. Some families have more than one patient and their features are separated from each other by comma

Family	Mutation	Zygosity	Age at onset (yrs)	Loss of ambulation (yrs)	Calf hypertrophy	Ankle contractures	Winging scapulae	Scoliosis	Lordosis	Serum CK (U/L)	Muscle biopsy	EMG with myopathic features
CAPN3												
F1	c.291C>A	Homo	19	ambulant at age 28	No	NA	Yes	NA	Yes	14000	NA	Myopathic pattern
F2	c.380G>A	Homo	5	No	Yes	No	Yes	No	Yes	4,092	MD	NA
F3	c.550delA	Homo	6, 6	30, Ambulant at age 28	NA, NA	No, No	NA, NA	NA, NA	NA, NA	922	MD	Yes
F4	c.567delG	Homo	6, 13	19, 23, Ambulant at age 11	No, No, Yes	No	Yes	NA, Yes, No	Yes, No, No	12000, 15070	NA	Myopathic disorder
F5	c.946-2A>G	Homo	12, 18	27, 26, Ambulant at age 25	No	No, NA	Yes	No	No	NA	NA	Yes
F6	c.956C > T/c.2257 delGinsAA	Compound hete	23	Ambulant at age 58	Slight	No	Yes	No	No	418	NA	Sever myopathic process
F7	c.1714C>T/c.2311G>A	Compound hete	11, 16	Ambulant at age 25,31	No	No, Yes, Yes, Yes	No	No	Yes, No, No, No	283,721	MD, NA	Yes, Yes, Yes, NA
F8	c.1894 A>T	Homo^a^	3, 4	NA^b^, No	No	No	No, Yes	NA, No	No, NA	NA	MD	Myopathic disorder
F9	c.2105C>T	Homo	9	Ambulant at age 24 and 29	Slight, No	NA, No	Yes, Slight	Yes, Slight	NA, No	NA	NA	NA
F10	c.2105C>T	Homo	8, 15	15,Ambulant at age of 19	No	Yes	Yes	No	Yes	5182-10,580	MD^c^	Yes
F11	c.2105 C>T/ c.380G>A	Compound hete^d^	12, 13	15, Ambulant at age 19	3,985	NA	Yes, NA	NA	Yes, No	2888-7,120	NA	Yes
F12	c.2243G>A	Homo	5-9	26, ambulant at age 25	No	No	Yes	No	Yes	558-1783	MD	Myopathic disorder
F13	c.2254-2256delAAC	Homo	10, 16	30, 30, Ambulant at age 22-41	NA	NA	NA	NA	NA, NA, Yes, NA	NA	NA	NA
F14	c.2373C>T	Homo	12	-	NA	+	+	+	+	2498	MD	Yes
F15	c.2380+2T>G	Homo	6, 11	23,25	Yes	Yes	No, Yes, Yes	No, Yes, Yes	No, Yes, Yes	NA	MD	NA
F16	-	-	1	Ambulant at age 11	No	No	No	No	No	1004	MD	No
F17	-	-	10,10,10,11	32,Ambulant at ages19,11,9	No,Yes,Yes,NA	NA	NA	NA	NA	NA	NA	NA
DYSF												
F18	c.(1053+1_1054-1)_(1397+1_1398-1)del	Homo	22,14	27,24	No, No	Yes, Yes	No, No	No, No	No, Yes	3000,2368	Dysferlinopathy	Yes
F19	c.2419C>T	Homo	35,23	43, 25	Yes, Yes	No, No	No, No	No, No	No, No	4500	Dysferlinopathy	Yes,Yes
F20	c.2706dupC	Homo	15	Ambulant at age 20	Yes	No	No	No	No	11726	Dysferlinopathy	Yes
F21	c.2706dupC	Homo	18	Ambulant at age 21	No	No	No	Yes	Yes	12000	Dysferlinopathy	Yes,BMD^e^
F22	c.3112C>T	Homo	19	29, 28	No, No	NA^1^, NA	Yes, No	No, Yes	Yes, Yes	3900	Dysferlinopathy	Yes
F23	c.3225delT	Homo	13,19	29, 30	No, No	No, No	No, No	No, No	No, No	4099, 6506	LGMD	Yes,Yes
F24	c.4639-1G>A	Homo	19,21,24	30,33,35	No, No,No	No, No,NA	No,No, No	No,No	No , Yes,Yes	3900, 2356,4500	Dysferlinopathy	Yes
F25	c.5633T>C	Homo	22, NA	Ambulant at ages of 33, 33	No, No	No, No	No,No	No, No	Yes,Yes	4586, 3850	Myositis	Chronic myopathy in lower limbs, Myoshi myopathy
F26	c.5804C>T	Homo	20,17,18	44, Ambulant at ages of 33 and 28	No, No, No	Yes, No, No	Yes, No, No	Yes, Yes, No	Yes, Yes,Yes	3000	LGMD	LGMD
SGCA												
F27	c. 319–329 delGCCTACAATCG	Homo	10,7,5	14,12,7	No,Yes,No	Yes,Yes,Yes	Yes,Yes,No	Yes,Yes,No	Yes,Yes,No	NA^b^, NA,16428	NA, NA ,Alpha sarcoglycanopathy	NA,Yes, Yes
F28	c.427C>A	Homo	6	-	Yes	NA	NA	NA	NA	13003	NA	NA
F29	c.687-688delTC	Homo	5	10	NA	NA	NA	NA	NA	5570	NA	DMD
SGCB												
F30	c.-10_16dup26	Homo	Normal at age 10,17	NA, Ambulant at age 17	Yes, Yes	No, No	No, Yes	No, Yes	No, Yes	6670, 1600	MD	Yes
F31	c.(33+1_34-1) _ (243+1_244-1) del	Homo	3,6	8,13	NA	Yes,Yes	Yes, Yes	No, Yes	Yes, Yes	10824	Beta sarcoglycanopathy	NA
F32	c.(33+1_34-1) _ (243+1_244-1) del	Homo	7,9,9,9	Ambulant at ages of 9 and 9,wheelchair bound at 13 and 10	NA	No,No,No, Yes	No,No, No,No	NA,No,No,Yes	Yes,Yes, Yes, Yes	9360	Beta sarcoglycanopathy	Yes
F33	c.(33+1_34-1) _ (243+1_244-1) del	Homo	5	NA	No	NA	No	NA	NA	8500	NA	Yes
F34	c.(33+1_34-1) _ (243+1_244-1) del	Homo	2,2	6,Ambulant at age 4	NA	Yes,NA	No,No	No,No	No,No	NA	NA	NA
F35	c.(33+1_34-1) _ (243+1_244-1) del	Homo	9,9	13,12	NA	Yes,Yes	Yes,Yes	Yes,Yes	Yes,Yes	840	NA	Yes
F36	c.(33+1_34-1) _ (243+1_244-1) del	Homo	2	Ambulant at age 8	Yes	NA	NA	NA	NA	9582	Beta sarcoglycanopathy	Yes
F37	c.(33+1_34-1) _ (243+1_244-1) del	Homo	5,6	11,14	No, No	No,NA	Yes,Yes	Yes, No	No, No	14500,11200	Beta sarcoglycanopathy	NA
F38	c.(33+1_34-1) _ (243+1_244-1) del	Homo	7	Ambulant at age 9	No	No	No	No	NA	7800	MD	Yes
F39	c.622-1G>C	Homo	NA	NA	NA	NA	NA	NA	NA	12395,23490,33450	Beta, Gamma sarcoglycanopathy	Yes
F40	c.753+1G>A	Homo	8	Ambulant at age 11	No	NA	NA	NA	NA	13600	NA	Yes, DMD

^a^Homozygote

^b^Not available

^c^Muscular Dystrophy

^d^Compound heterozygote

^e^Becker Muscular Dystrophy

### Mutation analysis

The exons and intron-exon boundaries of the relevant genes in families whose patients had homozygous haplotypes were sequenced, and the causative mutations were found in 38 out of 40 families. Totally 38 mutations were identified in *CAPN3* (*n* = 15), *DYSF* (*n* = 9), *SGCB* (*n* = 11), and *SGCA* (*n* = 3). All detected mutations are shown in Table 1. Five out of 38 mutations were novel (Table 2). They were evaluated using different software tools such as DANN, Human Splicing Finder (HSF), Functional Analysis through Hidden Markov Models (FATHMM), Genomic Evolutionary Rate Profiling (GERF), and mutation taster. DANN is a pathogenicity scoring methodology, and it ranges from 0 to 1, with 1 given to the variants predicted to be the most damaging. FATHMM is a high-throughput web-server capable of predicting the functional consequences of coding and non-coding variants. GERP is a conservation score, and it ranges from − 12.3 to 6.17. Score 6.17 is the most conserved. All variants’ pathogenicity was interpreted according to the ACMG guideline (Table 2).

**Table 2:** Novel variants observed in our patients

Family	Gene name	Mutation at DNA level	Mutation at protein level	Intron/exon number	DANN	HSF	FATHMM	GERP	Mutation taster	Zygosity	ACMG interpretation
F24	DYSF	c.4639-1G > A	–	42	0.99	Most probably affecting splicing	Damaging	5.36	Disease causing	Homo	Pathogenic (PVS1, PM2, PP3)
F28	SGCA	c.427C > A	p.His143Asn	5	0.98	–	Damaging	4.3499	Disease causing	Homo	Likely pathogenic (PS3, PM2, PP3, PP4)
F29	SGCA	c.687-688delTC	p.Leu230Valfs*13	6	–	–	–	5.1399	Disease causing	Homo	Pathogenic (PVS1, PM2, PP4)
F39	SGCB	c.622-1G > C	–	5	0.99	Most probably affecting splicing	Damaging	5.32	Disease causing	Homo	Pathogenic (PVS1, PM2, PP3, PP4)
F40	SGCB	c.753 + 1G > A	–	5	0.99	Most probably affecting splicing	Damaging	5.1199	Disease causing	Homo	Pathogenic (PVS1, PM2, PP3, PP4)

### Muscle biopsy studies

Muscle biopsy studies in calpainopathy are not specific and ranging from mild to severe dystrophic changes. Also besides, immunohistochemical markers are usually unreliable [15].

In dysferlinopathies, almost all fibers are stained with antibodies against dystrophin 1, 2, and 3, merosin, β-spectrin, and α, β, and γ sarcoglycans; but the muscle fibers are looked completely deficient against dysferlin antibody. In sarcoglycanopathies, labeling with all antibodies except for sarcoglycans was observed. Table 1 presents the results.

#### CAPN3

Affected members of 17 out of 60 families showed runs of homozygosity in the *CAPN3* gene, but causative mutations were found in 15 families. All of the families except for families F11, F7, and F6 had homozygous mutations. The mentioned families showed compound heterozygous mutations (Table 1). Eighteen mutations were identified in our patients, including ten missenses, three splicings, three deletions, one nonsense, and one deletion/insertion mutations. The most frequent mutations were found to be c.2105C > T and c.380G > A, in which the c.2105C > T mutation was in the homozygous state in two patients and compound heterozygous in one patient, and the c.380G > A mutation detected in homozygous and in compound heterozygous in one patient.

#### DYSF

Eight different mutations were found in nine families, in which two out of them, F20 and F21, who were from Lurs of Boyer-Ahmad revealed common haplotype and mutation in the *DYSF* gene [16]. We identified two deletions, two duplications, two missenses, two nonsenses, and one splicing mutations in this gene. One of the mentioned mutations, c.4639-1G > A, which resides in the intron 42, has not been previously reported. According to the HSF tool, it may alter the wild type acceptor site and activate an intronic cryptic acceptor site, which potentially can alter splicing. Its score in other in silico tools are as shown in Table 2.

#### SGCA

Three families had homozygous haplotypes in their affected members, and three mutations have been identified (Table 1), two deletion, and one missense mutations, in which two out of them were novel (Table 2). The deletion mutation, c.687-688delTC (p.Leu230Valfs*13), which is a frameshift one was seen in the homozygous state in the patient of the family 29 (F29). This mutation resides in the extracellular domain of the protein.

The other novel mutation was a missense one, changing amino acid histidine to asparagine in codon 143, p.His143Asn, which is also located in the extracellular domain of the protein (Table 2).

#### SGCB

Eleven families had mutations in the *SGCB* gene including two splicing, one duplication, and eight deletion mutations. Two splicing mutations were not previously reported in different mutation databases. One of them was c.753 + 1G > A that can disturb wild type donor site of splicing based on the HSF tool. Another one was c.622-1G > C which may disrupt the wild type acceptor site of splicing. The DANN score for both mutations was 0.99, the GERP score was almost 5, FATHMM results were “damaging” and mutation taster outcomes were “disease-causing”.

**Fig. 1:**
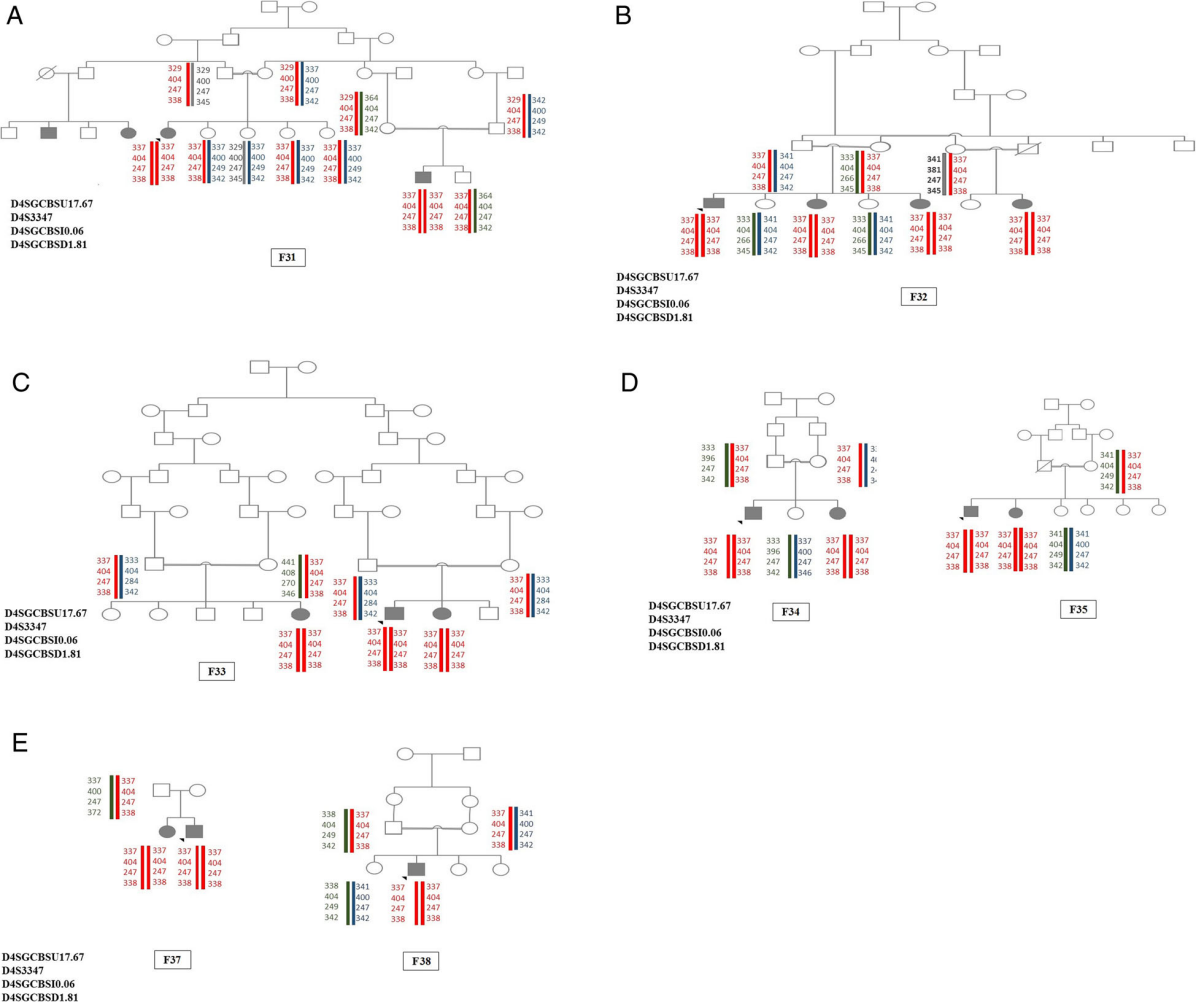
(**a**-**e**): Haplotypes of families with a homozygous deletion of exon 2. STR markers used for the *SGCB* gene are shown in each figure. Some markers have not been previously reported and we chose new names for them. U and D mean upstream and downstream respectively. The numbers denote the distance from the gene (e.g. 8.05 × 105 base pairs)

Eight families, F31 to F38, from south-east of Iran showed the same haplotype and same mutation in the *SGCB* gene. The haplotypes are shown in Fig. 1a-e. Performing PCR to sequence the whole *SGCB* gene revealed that all exons except exon 2 yield amplification products in the affected individuals. We repeated the PCR of this exon with several primer pairs, various annealing temperatures, and cycle numbers, but it did not yield any amplification product suggesting a possible deletion of this exon. Further analysis of the patients’ DNA has shown that the multiple primer sets flanking exon 2 failed to produce PCR product. More analysis using the MLPA technique confirmed the deletion of the exon 2, c. (33 + 1_34–1)_(243 + 1_244–1) del, of the *SGCB* gene in the patients (Additional file 1 Figure S1, Additional file 2: Figure S2, and Additional file 3: Figure S3). The mutation results in discarding codon 12 to 81, which leads to eliminating the large part of the cytoplasmic and transmembrane domains of the protein, and this mutation accounts for the most prevalent one in the *SGCB* gene in our studied population.

### First report of allelic heterogeneity in a consanguineous LGMD family

**Fig. 2:**
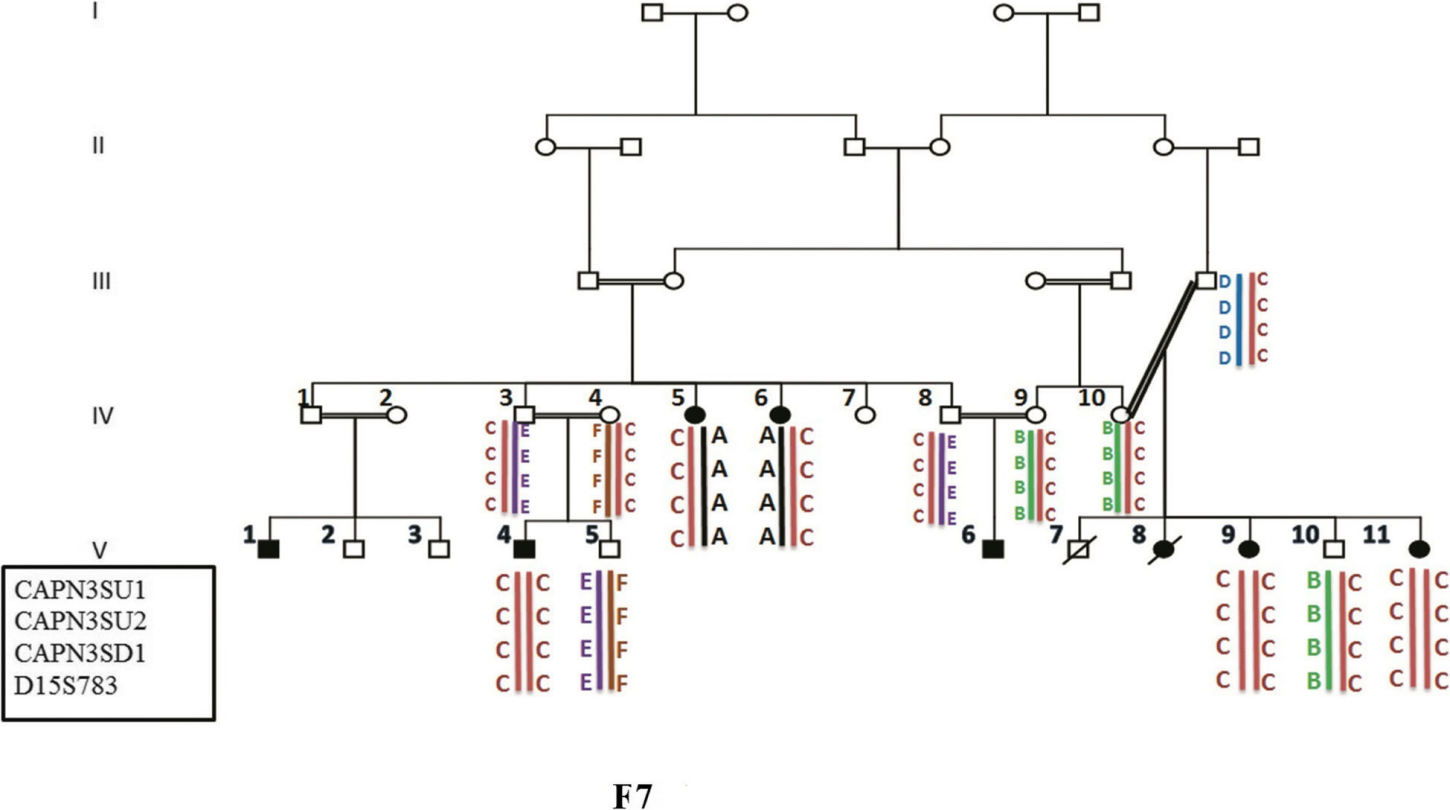
Autozygosity mapping in family P9 which showed allelic heterogeneity. Patients V4, V9 and V11 showed homozygous haplotypes, haplotypes C. Patients IV5 and IV6 of this family showed compound heterozygous haplotypes (haplotype A/C) for this gene

Family F7 in this study had eight affected individuals. Patients V4, V9, and V11 showed homozygous haplotypes, haplotypes C, for the gene *CAPN3*, which raise the possibility of co-segregating of the disease phenotype with *CAPN3* gene. Patients IV5 and IV6 of this family showed compound heterozygous haplotypes (haplotype A/C) for this gene (Fig. 2). All 24 exons and exon-intron boundaries of the *CAPN3* gene were sequenced. Patients V4, V9, and V11 showed the homozygous mutation of c.1714C > T in exon 13, which was seen in heterozygous form in patients IV5 and IV6. It raised the possibility of segregating of this mutation with haplotype C. This mutation caused the substitution of arginine to tryptophan at residue 572 (Arg572Trp). The other mutation which was seen in heterozygous form in patients IV5 and IV6, was c.2311G > A in exon 22, changing alanine to Threonine. This mutation is segregated with haplotype A. Both mutations were checked in all family members.

## Discussion

### Prevalence of detected mutations in this study

Due to limited epidemiology data and low incidence of LGMDs, few reports have been published about the approximate prevalence of each subtype. In a large cohort study in 4656 clinically suspected-LGMD patients across the US, the diagnosis was established in 27%, in which calpainopathy and dysferlinopathy were the most prevalent subtypes [15, 17, 18]. In a study performed on 20 Turkish patients, calpainopathy was the most prevalent form, and dysferlinopathy was the least form of LGMDs, and among sarcoglycanopathies, the mutation in the *SGCG* gene was the most common and in the *SGCA* gene was the least common form [19]. In another study conducted by Fanin et al*,* LGMDR1 calpain-3 related was the most prevalent form in Italian patients, and sarcoglycanopathies were the second most common type, in which alpha sarcoglycanopathies were the most frequent forms [20]. A study on 105 Chinese patients, Showed that the most common subtype was LGMDR2 dysferlin related, and LGMDR3 α -sarcoglycan related is the most frequent type of sarcoglycanopathies [21]. In research by Okizuka et al., the incidence of LGMDR5 γ -sarcoglycan-related was estimated to be 1 per 560,000 in the Japanese people [22]. According to a study by Pathak et al., the most common form of LGMD in the Indian population was LGMDR1 calpain3-related [23]. The most common causes of LGMD in Australia were calpainopathy and dysferlinopathy [24]. Duno et al. demonstrated that calpainopathy was not a common cause of LGMD in Denmark [25], and unexpectedly, LGMDR9 FKRP-related had the highest frequency amongall LGMDs in this country [26]. A study in Iranian affected individuals with sarcoglycanopathy showed that LGMDR4 β-sarcoglycan-related (LGMD2E) is the most common form of sarcoglycanopathies in Iran [2].

In the present study, 38 out of 60 families who were suspected of being affected by LGMDs had mutation(s) in *CAPN3, DYSF, SGCA*, and *SGCB* genes. The most prevalent form of LGMDs in our study was calpainopathy followed by sarcoglycanopathy in which beta-sarcoglycanopathy was the most common form. Exon 2 deletion in the *SGCB* gene was the most prevalent mutation in this study. Further studies can help us to determine the frequency of different types of LGMDs and mutations in the Iranian population.

In spite of observing homozygosity for the *CAPN3* gene in two families of F16 and F17, the causative mutation was not found. It might be because of incidental finding of homozygosity in these families, or the mutation may reside in deep intronic sequences or in regulatory elements that are not sequenced by Sanger sequencing in the present study.

### Evidence of a possible founder effect in our studied patients

Two unrelated families, F20 and F21, had the same mutation, c.2706dupC, and haplotype for the *DYSF* gene; since they had the same ethnicity and were from Lur, this observation may be suggestive of a possible founder effect [16]. To our knowledge, this variant has not been previously reported in Iranian population but it was reported by Cacciottolo et al. in Italy in 2011 [27]. More sample size is needed to confirm the founder effect.

We also found eight families with the same haplotype and same mutation. The mutation leads to the deletion of exon 2. This mutation is a pathogenic one according to the ACMG guideline and causes elimination of anchor domain of the SGCB protein which may cause deleterious effect on the assembly of sarcoglycan complex. The families were from the south-east of Iran and the Baloch ethnic group. In another study by Alavi et al., it was shown that almost 85% (12 out of 14) of their LGMD2E patients had a deletion that encompassed whole exon 2 in *SGCB* gene [2]; 10 out of 12 of their studied individuals with this deletion were from south and south-east of Iran; haplotype analysis based on three Single nucleotide polymorphisms (SNP) markers were also suggestive of a possible founder effect in this region in Iran; and can be beneficial in mutation screening of the LGMD2 diagnosed patients from this area.

Further studies with more sample size and additional markers are required to establish a probable founder effect.

### Reporting of five novel mutations

We observed a novel deletion mutation of c.687-688delTC in the *SGCA* gene, which results in producing a truncated protein and elimination of downstream part of the protein including cytoplasmic and transmembrane and some parts of the extracellular domain of the SGCA protein. According to the ACMG guideline, it is a pathogenic variant.

Another novel mutation, c.427C > A (p.His143Asn), results in a smaller amino acid, which might lead to loss of interactions [28]. Segregation analysis in this family has done in all family members. Since this variant is absent from controls in Exome Sequencing Project, 1000 Genomes Project, or Exome Aggregation Consortium (PM2), multiple lines of in silico analysis support a deleterious effect on the gene (PP3), patient’s phenotype is highly specific for the disease (PP4), and the patient has raised CpK concentration of about 13,003 (U/L) which it can functionally explain the deleterious effect of the mutation (PS3); the variant of c.427C > A in *SGCA* gene is a likely pathogenic variant.

Two novel mutations which have been observed in *SGCB* gene, c.753 + 1G > A and c.622-1G > C, are pathogenic according to the ACMG interpretation guideline because null variants like canonical ±1 or 2 splice sites provide a very strong evidence of pathogenicity for a variant, the other indications are as follows: PM2, PP3, PP4 which have been described previously.

We detected a mutation in the *DYSF* gene, c.4639-1G > A, which has not been previously reported. This mutation most probably affects splicing and according to the ACMG guideline is a pathogenic one (PVS1, PM2, PP3).

### Unexpected allelic heterogeneity in *CAPN3* gene within a single large consanguineous family

Both observed mutations in the family F7 were previously reported [29–31] and are likely pathogenic according to the ACMG guideline (PM1, PM2, PM5, PP3, PP4, PP5 for c.1714C > T, and PM1, PM2, PP3, PP4 for c.2311G > A).

It’s a general rule that in rare recessive conditions, autozygous mutations are more likely to be causative than compound heterozygous ones [32], but it has been shown that in highly consanguineous populations, consanguinity has a powerful effect in the occurrence of many rare diseases than founder effect and it results in allelic heterogeneity even in genetically isolated populations (or an extended family) [33]. Locus heterogeneity in LGMD has been previously reported in a family from Tunis whose two cousins were affected by LGMDR5 γ -sarcoglycan-related and LGMDR3 α -sarcoglycan-related in a consanguineous family [34]. What we saw in the present study was allelic heterogeneity within a highly consanguineous Iranian family which was the first report of allelic heterogeneity in the LGMD, and for such a rare disease, it can make us pay more attention to the difficulty of genetic counseling in inbred populations. We have to be more careful about genetic counseling of families with multiple consanguineous loops when homozygosity in one mutated allele is expected. Autozygosity mapping in such families can be helpful to display genetic heterogeneity, both locus and allelic [35].

## Conclusion

This study could shed light on the genetic cause of 112 Iranian patients in 38 unrelated families carrying 31 different kinds of mutations. Investigation in other families is going on. Calpainopathy was the most prevalent subtype in our studied sample. We identified five novel pathogenic variants that enrich human genetic mutation databases. This study can expand our knowledge about the genetic spectrum of LGMD in Iran.

## Methods

### Subjects

Sixty families suspicious of being affected by LGMDs were referred to Kawsar Human Genetics Research Center (KHGRC). Prior to sampling, genetic counseling was performed, and Informed consent from all families was obtained. The project was approved by the ethical committee of the Pasteur Institute of Iran (No: 91/0201/10425).

### Muscle biopsy

Fresh muscle specimens were taken from subjects and quickly frozen in isopentane cooled by liquid nitrogen. The specimens were evaluated using the conventional panel of histochemical stains including H&E, Gomori Modified Trichrome, Congo red, PAS (periodic Acid-Schiff), Oil red O (ORO), NADH-tetrazolium reductase (NADH-TR), succinate dehydrogenase (SDH), cytochrome C oxidase (COX), Modified SDH/COX double stain and ATPase (adenosine triphosphatase) × 3. Immunohistochemical staining was performed using mouse monoclonal antibodies against dystrophin (1–3, and), mouse monoclonal antibodies against SGs (α, γ, and β), rabbit monoclonal antibodies against dysferlin, mouse monoclonal antibodies against β-spectrin, and mouse monoclonal antibodies against merosin as primary antibody, and HRP-tagged as the secondary antibody (Novolink, US). Beta-spectrin was applied as a positive control.

### Autozygosity mapping and mutation analysis

Genomic DNA was extracted from peripheral blood using the salting out procedure (Miller et al. 1988). Four STR markers for each candidate genes of LGMDR1 calpain3-related to LGMDR6 δ-sarcoglycan-related were selected using Map viewer (http://www.ncbi.nlm.nih.gov/projects/mapview), TRF (http://tandem.bu.edu/trf/trf.html) [36] and SERV (http://www.igs.cnrsmrs.fr/SERV/) [37] online tools. These 24 STRs were applied in two sets of multiplex PCR using Labeled primers. DNA sequencing, interpretation, and fragment analysis were done as previously described [14].

### MLPA

The MLPA test was performed on eight probands using the SALSA MLPA P116 SGC probe mix (for all sarcoglycan’s genes) and SALSA MLPA EK1 reagent kit (MRC Holland-Amsterdam-the Netherland) under the manufacturer’s protocols [38]. This kit was used in those patients who their exon 2 amplification of the *SGCB* gene fail to produce any product.

### In silico analysis

Predicting variant effects on protein structure was performed using six different in-silico tools, including SIFT [39], CADD [40], Poly Phen-2 [41], HSF [42], PANTHER [7], and mutation taster [43]. All variants were interpreted according to the American College of Medical Genomics and Genetics (ACMG) guideline [44].

## Supplementary information

**Additional file 1 : Figure S1**. MLPA result of a normal control individual

**Additional file 2 : Figure S2**. MLPA result of an individual carrying exon 2 deletion of the *SGCB* gene (hetero deletion). This figure shows the result of patients’ parents with a homozygous deletion of the mentioned exon.

**Additional file 3 : Figure S3**. MLPA result of a patient with a homozygous deletion of the exon 2 of the *SGCB* gene.

## Data Availability

The datasets used and/or analysed during the current study are available from the corresponding author on reasonable request.

## Abbreviations

ACMG: American College of Medical Genomics and Genetics
COX: Cytochrome C oxidase
FATHMM: Functional analysis through hidden markov models
GERF: Genomic evolutionary rate profiling
HSF: Human splicing finder
KHGRC: Kawsar human genetics research center
NADH-TR: NADH-tetrazoliumreductase
ORO: Oil red O
PAS: Periodic Acid-Schiff
SDH: Succinate dehydrogenase

## Footnotes

1 Dysferlinopathy characterized by two principal phenotypes including Miyoshi myopathy and LGMD2B

## References

[1] Arzani M, Rezaei H, Moghadasi AN (2018). Association of limb-girdle muscular dystrophy with multiple sclerosis: a case report. Caspian J Int Med.

[2] Alavi A, Esmaeili S, Nilipour Y, Nafissi S, Tonekaboni SH, Zamani G (2017). LGMD2E is the most common type of sarcoglycanopathies in the Iranian population. J Neurogenet.

[3] Nigro V, Savarese M, Nigro V, Savarese M. Genetic basis of limb-girdle muscular dystrophies: the 2014 update. Acta Myol. 2014;33:1–122014. 1–12. PMC4021627 24843229

[4] Liewluck Teerin, Milone Margherita (2018). Untangling the complexity of limb‐girdle muscular dystrophies. Muscle & Nerve.

[5] Gallardo E, Saenz A, Illa I (2011). Limb-girdle muscular dystrophy 2A. Handb Clin Neurol.

[6] Kramerova I, Beckmann JS, Spencer MJ (2007). Molecular and cellular basis of calpainopathy (limb girdle muscular dystrophy type 2A). Biochim Biophys Acta (BBA) - Mol Basis Dis.

[7] Pantoja-Melendez CA, Miranda-Duarte A, Roque-Ramirez B, Zenteno JC (2017). Epidemiological and molecular characterization of a Mexican population isolate with high prevalence of limb-girdle muscular dystrophy type 2A due to a novel Calpain-3 mutation. PLoS One.

[8] Bansal D, Campbell KP (2004). Dysferlin and the plasma membrane repair in muscular dystrophy. Trends Cell Biol.

[9] Chiu Y-H, Hornsey MA, Klinge L, Jørgensen LH, Laval SH, Charlton R (2009). Attenuated muscle regeneration is a key factor in dysferlin-deficient muscular dystrophy. Hum Mol Genet.

[10] Kerr JP, Ziman AP, Mueller AL, Muriel JM, Kleinhans-Welte E, Gumerson JD (2013). Dysferlin stabilizes stress-induced Ca&lt;sup&gt;2+&lt;/sup&gt; signaling in the transverse tubule membrane. Proc Natl Acad Sci.

[11] de Luna N, Gallardo E, Soriano M, Dominguez-Perles R, de la Torre C, Rojas-García R (2006). Absence of Dysferlin Alters Myogenin Expression and Delays Human Muscle Differentiation “in Vitro”. J Biol Chem.

[12] Politano L, Nigro V, Passamano L, Petretta V, Comi LI, Papparella S (2001). Evaluation of cardiac and respiratory involvement in sarcoglycanopathies. Neuromuscul Disord.

[13] Vahidnezhad H, Youssefian L, Jazayeri A, Uitto J (2018). Research techniques made simple: genome-wide Homozygosity/Autozygosity mapping is a powerful tool for identifying candidate genes in autosomal recessive genetic diseases. J Invest Dermatol.

[14] Mojbafan M, Tonekaboni SH, Abiri M, Kianfar S, Sarhadi A, Nilipour Y (2016). Linkage study revealed complex haplotypes in a multifamily due to different mutations in CAPN3 gene in an Iranian ethnic group. J Mol Neurosci.

[15] Strafella C, Campoli G, Galota RM, Caputo V, Pagliaroli G, Carboni S (2019). Limb-girdle muscular dystrophies (LGMDs): the clinical application of NGS analysis, a family case report. Front Neurol.

[16] Mojbafan M, Tina S, Motlagh FZ, Surguchov A, Nilipour Y, Zeinali S. Genetic variability in Iranian limb-girdle muscular dystrophy type 2B patients: an evidence of a founder effect. Mol Genet Genomic Med. 2019;7(12):e1029. 10.1002/mgg3.1029 PMC6900382 31693312

[17] Liu W, Pajusalu S, Lake NJ, Zhou G, Ioannidis N, Mittal P (2019). Estimating prevalence for limb-girdle muscular dystrophy based on public sequencing databases. Genet Med.

[18] Nallamilli BRR, Chakravorty S, Kesari A, Tanner A, Ankala A, Schneider T (2018). Genetic landscape and novel disease mechanisms from a large LGMD cohort of 4656 patients. Ann Clin Transl Neurol.

[19] Dincer P, Leturcq F, Richard I, Piccolo F, Yalnizoglu D, de Toma C (1997). A biochemical, genetic, and clinical survey of autosomal recessive limb girdle muscular dystrophies in Turkey. Ann Neurol.

[20] Fanin M, Nascimbeni AC, Aurino S, Tasca E, Pegoraro E, Nigro V (2009). Frequency of LGMD gene mutations in Italian patients with distinct clinical phenotypes. Neurology..

[21] Yu M, Zheng Y, Jin S, Gang Q, Wang Q, Yu P (2017). Mutational spectrum of Chinese LGMD patients by targeted next-generation sequencing. PLoS One.

[22] Okizuka Y, Takeshima Y, Itoh K, Zhang Z, Awano H, Maruyama K (2010). Low incidence of limb-girdle muscular dystrophy type 2C revealed by a mutation study in Japanese patients clinically diagnosed with DMD. BMC Med Genet.

[23] Pathak P, Sharma MC, Sarkar C, Jha P, Suri V, Mohd H (2010). Limb girdle muscular dystrophy type 2A in India: a study based on semi-quantitative protein analysis, with clinical and histopathological correlation. Neurol India.

[24] Lo HP, Cooper ST, Evesson FJ, Seto JT, Chiotis M, Tay V (2008). Limb-girdle muscular dystrophy: diagnostic evaluation, frequency and clues to pathogenesis. Neuromuscul Disord.

[25] Duno M, Sveen ML, Schwartz M, Vissing J (2008). cDNA analyses of CAPN3 enhance mutation detection and reveal a low prevalence of LGMD2A patients in Denmark. Eur J Human Genet.

[26] Sveen ML, Schwartz M, Vissing J (2006). High prevalence and phenotype-genotype correlations of limb girdle muscular dystrophy type 2I in Denmark. Ann Neurol.

[27] Cacciottolo M, Numitone G, Aurino S, Caserta IR, Fanin M, Politano L (2011). Muscular dystrophy with marked Dysferlin deficiency is consistently caused by primary dysferlin gene mutations. Eur J Hum Genet.

[28] Venselaar H, Te Beek TA, Kuipers RK, Hekkelman ML, Vriend G (2010). Protein structure analysis of mutations causing inheritable diseases. An e-Science approach with life scientist friendly interfaces. BMC bioinformatics.

[29] Fattahi Z, Kalhor Z, Fadaee M, Vazehan R, Parsimehr E, Abolhassani A (2017). Improved diagnostic yield of neuromuscular disorders applying clinical exome sequencing in patients arising from a consanguineous population. Clin Genet.

[30] Blazquez L, Azpitarte M, Saenz A, Goicoechea M, Otaegui D, Ferrer X (2008). Characterization of novel CAPN3 isoforms in white blood cells: an alternative approach for limb-girdle muscular dystrophy 2A diagnosis. Neurogenetics..

[31] Fanin M, Fulizio L, Nascimbeni A, Spinazzi M, Piluso G, Ventriglia V (2004). Molecular diagnosis in LGMD2A: mutation analysis or protein testing?. Hum Mutat.

[32] King RA, Rotter JI, Motulsky AG. The genetic basis of common diseases. New York & Oxford; Oxford University Press; 2002.

[33] Aldahmesh MA, Abu-Safieh L, Khan AO, Al-Hassnan ZN, Shaheen R, Rajab M (2009). Allelic heterogeneity in inbred populations: the Saudi experience with Alstrom syndrome as an illustrative example. Am J Med Genet A.

[34] Lezirovitz K, Pardono E, de Mello Auricchio MT, de Carvalho ESFL, Lopes JJ, Abreu-Silva RS (2008). Unexpected genetic heterogeneity in a large consanguineous Brazilian pedigree presenting deafness. Eur J Hum Genet.

[35] Miano MG, Jacobson SG, Carothers A, Hanson I, Teague P, Lovell J (2000). Pitfalls in homozygosity mapping. Am J Hum Genet.

[36] Benson G (1999). Tandem repeats finder: a program to analyze DNA sequences. Nucleic Acids Res.

[37] Legendre M, Pochet N, Pak T, Verstrepen KJ (2007). Sequence-based estimation of minisatellite and microsatellite repeat variability. Genome Res.

[38] Wildforster V, Dekomien G (2009). Detecting copy number variations in autosomal recessive limb-girdle muscular dystrophies using a multiplex ligation-dependent probe amplification (MLPA) assay. Mol Cell Probes.

[39] Sim N-L, Kumar P, Hu J, Henikoff S, Schneider G, Ng PC (2012). SIFT web server: predicting effects of amino acid substitutions on proteins. Nucleic Acids Res.

[40] Rentzsch Philipp, Witten Daniela, Cooper Gregory M, Shendure Jay, Kircher Martin (2018). CADD: predicting the deleteriousness of variants throughout the human genome. Nucleic Acids Research.

[41] Adzhubei IA, Schmidt S, Peshkin L, Ramensky VE, Gerasimova A, Bork P (2010). A method and server for predicting damaging missense mutations. Nat Methods.

[42] Desmet FO, Hamroun D, Lalande M, Collod-Beroud G, Claustres M, Beroud C (2009). Human splicing finder: an online bioinformatics tool to predict splicing signals. Nucleic Acids Res.

[43] Schwarz JM, Cooper DN, Schuelke M, Seelow D (2014). MutationTaster2: mutation prediction for the deep-sequencing age. Nat Methods.

[44] Richards S, Aziz N, Bale S, Bick D, Das S, Gastier-Foster J (2015). Standards and guidelines for the interpretation of sequence variants: a joint consensus recommendation of the American College of Medical Genetics and Genomics and the Association for Molecular Pathology. Genet Med.

