# Supplementary figures and images for: Mutational spectrum of autosomal recessive limb-girdle muscular dystrophies in a cohort of 112 Iranian patients and reporting of a possible founder effect
Source: Orphanet J Rare Dis. 2020 Jan 14;15:14. doi: 10.1186/s13023-020-1296-x (PMC6961257; doi:10.1186/s13023-020-1296-x)

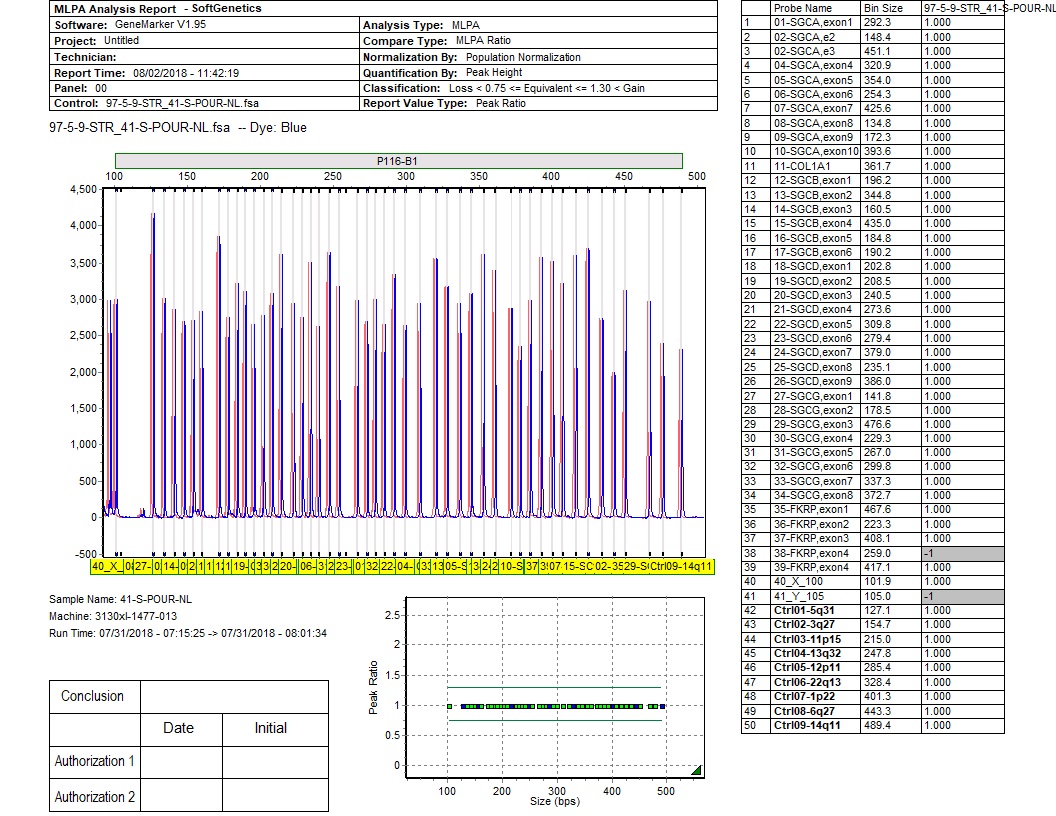

Supplement: Supplementary file 1 — Additional file 1 : Figure S1. MLPA result of a normal control individual [file 13023_2020_1296_MOESM1_ESM.jpg]

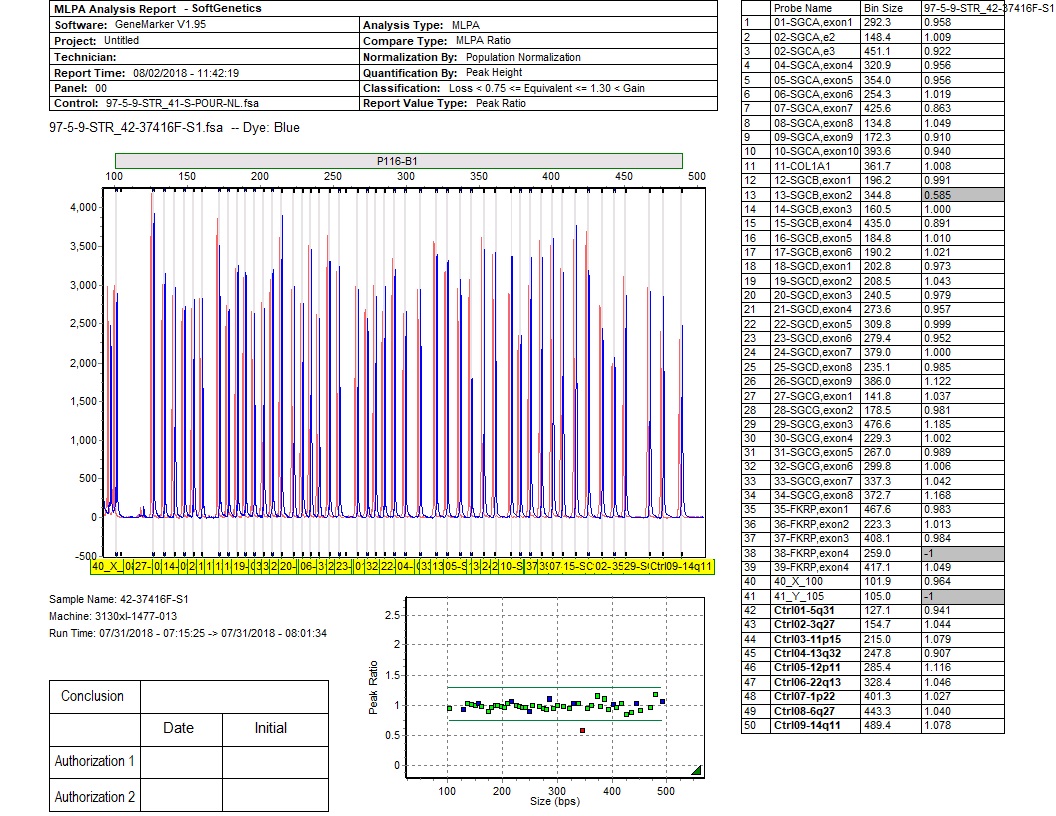

Supplement: Supplementary file 2 — Additional file 2 : Figure S2. MLPA result of an individual carrying exon 2 deletion of the SGCB gene (hetero deletion). This figure shows the result of patients’ parents with a homozygous deletion of the mentioned exon. [file 13023_2020_1296_MOESM2_ESM.jpg]

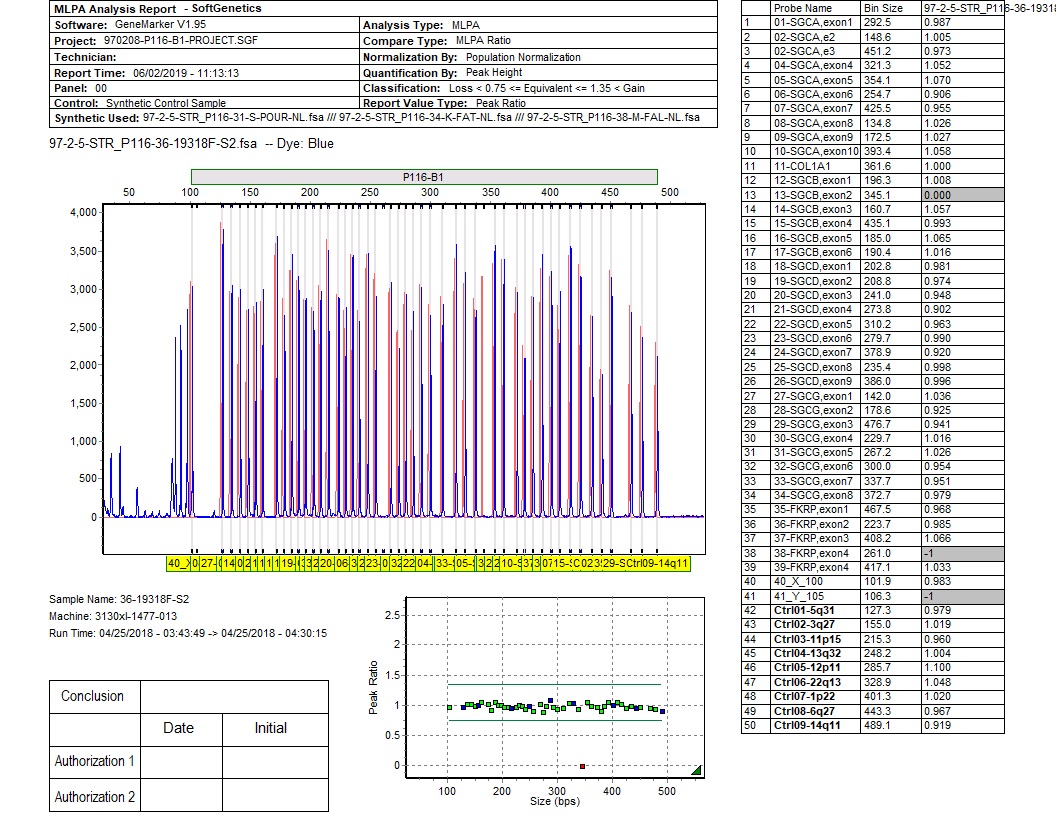

Supplement: Supplementary file 3 — Additional file 3 : Figure S3. MLPA result of a patient with a homozygous deletion of the exon 2 of the SGCB gene. [file 13023_2020_1296_MOESM3_ESM.jpg]
